# Multiple sclerosis and COVID-19: a northern China survey

**DOI:** 10.1007/s10072-024-07578-6

**Published:** 2024-05-09

**Authors:** Qian Guo, Tianwei Wang, Yusen Huang, Fangruyue Wang, Pingping Hao, Le Fang

**Affiliations:** 1 Department of Neurology, China-Japan Union Hospital of Jilin University, 126 Xiantai Street, Changchun, 130000 China; 2 Department of Radiology, China-Japan Union Hospital of Jilin University, Changchun, 130000 China; 3 The Third Bethune Hospital of Jilin University, Changchun, 130000 China

**Keywords:** Severe acute respiratory syndrome coronavirus type-2, Coronavirus disease 2019, Multiple sclerosis, Disease course, Disease-modifying therapy

## Abstract

**Background:**

There is insufficient data on severe acute respiratory syndrome coronavirus type-2 (SARS-CoV-2) infection in Chinese patients with multiple sclerosis (pwMS). This study aims to explore the manifestation of pwMS during the Coronavirus disease 2019 (COVID-19) pandemic and the effect of SARS-CoV-2 infection on the prognosis of MS in northern China.

**Methods:**

In this cross-sectional study, an online self-administered questionnaire and telephone interviews were conducted among pwMS of northern China. Clinical correlation of SARS-CoV-2 infection since the onset of the COVID-19 pandemic in northern China was analyzed.

**Results:**

164 patients with an average age of 38.9 ± 12.2 years were included, of which 57.3% had a disease course ≤ 5 years. 33.5% of the patients were COVID-19 vaccinated. 87.2% received disease-modifying therapy (DMT), and the average immunotherapy duration was 1.9 ± 1.6 years. 83.5% were SARS-CoV-2 infected, 14.6% reported worsening of their original condition after infection, and 5.1% had a relapse of MS. Shorter disease course was independently related to infection risk (*P* = 0.046), whereas increasing age was related to aggravated behavioral symptoms (*P* = 0.008). However, gender, vaccination, and DMT were not associated with susceptibility or poor prognosis.

**Conclusion:**

A shorter disease course is independently associated with an increased risk of SARS-CoV-2 infection, and age is associated with worsening disability. It seems to be safe and necessary to use DMT during the pandemic, however, the use of B cell-depletion agents should be approached with caution.

## Introduction

Coronavirus disease 2019 (COVID-19) is caused by severe acute respiratory syndrome coronavirus type-2 (SARS-CoV-2) [1]. Emerged in December 2019, the COVID-19 pandemic rapidly spread worldwide after impacting some regions, attracting close global attention [2]. Given the effect of SARS-CoV-2 on the human immune system, the relationship between autoimmune diseases and COVID-19 has become a focus of attention [3, 4].

MS is an autoimmune-mediated chronic demyelinating disease of the CNS [5, 6]. COVID-19 exhibits a high mortality rate among individuals with chronic diseases [7]. The enduring COVID-19 pandemic has raised concerns regarding infection risk and disease severity among individuals with immune system disorders. There is a lack of evidence to support a higher likelihood of patients with MS (pwMS) developing COVID-19 and having a worse prognosis compared to the general population. Certain studies have proposed that immunotherapy may potentially act as a protective factor against worse COVID-19 outcomes [8].

The prevalence and incidence of MS varies considerably across countries and regions. In Asian countries, MS is thought to be less prevalent compared to Western countries, with Asians having an 80 percent lower risk than Caucasians. While China is in a low-risk area for MS, recent years have seen an increase in both prevalence and incidence [9]. Based on data from a cohort of MS in Shandong province (located in northern China), it was shown that the incidence of MS increases with age, peaking at 15–29 and 30–44 years of age in male and female patients, respectively. The estimated prevalence in this study was 3.7 cases per 100,000 in men and 6.7 cases per 100,000 in women, resulting in an overall prevalence of 5.2 per 100,000 persons. The incidence of MS was 0.12/100,000 in men and 0.2/100,000 in women [10].

COVID-19 became widespread in northern China in December 2022. The effective genomic sequences of reported cases across different regions in China demonstrated the dominance of *Omicron BA.5.2* variant in the three provinces of northern China during the pandemic [11]. Before then, due to China’s lockdown policy, the SARS-CoV-2 infection rate among the Chinese population remained generally low. Subsequently, we conducted a cross-sectional study investigating the general conditions of pwMS, including gender, age, disease course, vaccination, and immunotherapy, on the manifestation and prognosis of pwMS during the COVID-19 pandemic. To our knowledge, this is the first reported survey on SARS-CoV-2 infection and its prognosis in northern Chinese pwMS after the lockdown was lifted.

## Patients and methods

### Study participants

This cross-sectional study included an online self-administered questionnaire sent to a cohort of registered pwMS in three neighboring provinces of northern China. The number of pwMS who were definitively diagnosed and registered in three neighboring provinces of northern China was 464.

On 1 January 2023, the investigator sent recruitment information to pwMS via the WeChat groups, including: the purpose of the study, information approved by the Ethics Committee, informed consent and 14 questions that could be answered using the self-administered questionnaire (Tool for Questionnaire Star, licensed). The questions were as follows: (1) age; (2) contact number; (3) gender; (4) place of origin; (5) date of birth; (6) diagnosis; (7) the drug used to control the disease (to prevent recurrence); (8) The duration the drug was taken (fill in it independently); (9) diagnosis time; (10) the number of COVID-19 vaccination shots received. (11) Whether the condition was stable before this pandemic, (12) whether infected with SARS-CoV-2, (13) whether the symptoms after infection are (multiple choices), and (14) whether the condition was aggravated after infection.

The study respected patients' willingness, and they were included in the study by scanning QR code to fill out questionnaire, only if they completed the questionnaire on their own initiative and provided a complete and comprehensive response. For any questions about filling out, we clarified them through point-to-point telephone contact.

A total of 164 pwMS completed the questionnaire, of which 137 (83.5%) were infected with SARS-CoV-2 and 20 (14.6%) felt that their original condition worsened after SARS-CoV-2 infection. Since then, we administered two supplementary questionnaires to the 20 patients with aggravated illness. The supplementary questionnaire contains two questions: (1) whether MS relapses and (2) the specific manifestations of the perceived disease aggravation, including behavioral, cognitive, psychological, and four other aspects.

If polymerase chain reaction or antibody test results are positive, the patient is diagnosed with SARS-CoV-2 infection [2]. Relapse of MS was defined as the presence of Ga-enhanced or T2 lesions on contrast-enhanced cranial MRI [6].

MS symptoms consist of four main aspects, which exacerbations are clearly defined as follows: 1). Behavioral manifestations, such as new-onset limb weakness, sensory abnormalities, ocular symptoms, ataxia, or exacerbations of the above-mentioned symptoms. 2). Cognitive manifestations, such as new-onset learning, memory deficits, delayed reaction time, or aggravations of the above-mentioned symptoms. 3). Psychological manifestations, such as new-onset depression, irritability, short-temperedness, euphoria, etc. or aggravations of the above-mentioned symptoms. 4). Other manifestations, such as new-onset bladder dysfunction or exacerbation [5, 12].

### Statistical analysis

SPSS25.0 was used to analyze the data. The Shapiro–Wilk test was used to test the normal distribution of the data. The classified data were tested by the χ^2^ test, χ^2^ correction test, and Fisher exact test, and the continuous data were tested by the t-test or rank sum test. Quantitative data were expressed as average and standard deviation (SD), while classified data were expressed as frequencies and percentages. Univariate and multivariate logistic regression models were used to evaluate the relationship between SARS-CoV-2 infection and the identified variables. We conducted multivariate stepwise logistic regression analysis with SARS-CoV-2 infection as the dependent variable and gender, age, disease course, and time of immunotherapy as independent variables. The results were expressed as an odds ratio (OR) and a 95% confidence interval (CI), *p* < 0.05 was considered statistically significant.

## Results

### General characteristics

As of 31 March 2023, a total of 164 patients completed the survey, which included 119 (72.2%) females with an average age of 38.9 ± 12.2 years. Of the respondents, 94 (57.3%) had a disease course of ≤ 5 years and 55 (33.5%) had been vaccinated against SARS-CoV-2. Most respondents, 143 (87.2%), had received DMT, and the average duration of immunotherapy was 1.9 ± 1.6 years.

### SARS-CoV-2 infection incidence and variables related to infection risk

Of the 164 respondents, 137 patients (83.5%) were confirmed to be infected with the SARS-CoV-2. Table 1 shows the differences between SARS-CoV-2-negative and SARS-CoV-2-positive patients overall.

**Table 1:** Differences between SARS-CoV-2-negative and SARS-CoV-2-positive patients overall

	SARS-CoV-2- negative (*N* = 137)	SARS-CoV-2- positive (*N* = 27)	*P* Value
Gender ^a^			
Male	40 (29.2)	5 (18.5)	0.256
Female	97 (70.8)	22 (81.5)	
Age, years ^b^	38 ± 11.3	41.6 ± 16.1	0.371
Drug type ^a^			
Unmedicated	15 (10.9)	3 (11.1)	0.174
Glucocorticoid drugs	3 (2.2)	0	
Siponimod or Fingolimod	51 (37.2)	10 (37.0)	
Ofatumumab	5 (3.6)	3 (11.1)	
Dimethyl fumarate	3 (2.2)	3 (11.1)	
Teriflunomide	58 (42.3)	8 (29.6)	
Rituximab	2 (1.5)	0	
The duration the drug was taken, years ^b^	1.9 ± 1.7	1.4 ± 1.4	0.056
≤ 1 ^a^	68 (49.6)	16 (59.3)	0.360
> 1 ^a^	69 (50.4)	11 (40.7)	
Vaccination ^a^			
Unvaccinated	91 (66.4)	18 (66.7)	0.980
Vaccinated, dosage ^a^	46 (33.6)	9 (33.3)	
1	5 (3.6)	0	0.674
2	20 (14.6)	6 (22.2)	
3	21 (15.3)	3 (11.1)	
Disease course, years ^a^			
≤ 5	82 (59.9)	12 (44.4)	0.026/0.028
5–10	33 (24.1)	8 (29.6)	
10–15	14 (10.2)	1 (3.7)	
> 15	8 (5.8)	6 (22.2)	

The course of MS was statistically significant to the infection risk of SARS-CoV-2 (*P* = 0.026). Based on this grouping, a significant discrepancy between disease courses that were ≤ 5 and those that were > 15 (*P* = 0.028) can be concluded through further multiple comparisons

*SARS-CoV-2* severe acute respiratory syndrome coronavirus type-2, *MS* multiple sclerosis

^a^ Values given as *N* (%)

^b^ Values given as mean ± standard deviation

Table 2 summarizes the various factors influencing SARS-CoV-2 infection risk in pwMS with Binary logistic regression analysis. The course of MS was statistically significant to the infection risk of SARS-CoV-2 (*P* = 0.026). There was a significant difference in the infection risk between patients with a course of ≤ 5 years and patients with a course of > 15 years (*P* = 0.028). Furthermore, a shorter course of disease is an independent risk factor for SARS-CoV-2 infection (OR = 1.57, 95% CI = 1.01–2.45, *P* = 0.046). The infection rate was higher in the group with a course of ≤ 5 years (87.2% ≤ 5 vs 57.1% > 15). Among patients with DMT, 119 (83.2%) were infected with SARS-CoV-2. SARS-CoV-2 infection risk did not differ significantly with patient gender, age, vaccination, immunotherapy, and immunotherapy time.

**Table 2:** Binary logistic regression analysis predicting the various factors influencing SARS-CoV-2 infection risk in pwMS

Predictors	SARS-CoV-2 infection risk			
	Odds Ratios	Std. Error	95% CI	*P* Value
Gender	2.18	0.56	0.73–6.46	0.161
Age	1.01	0.02	0.98–1.01	0.505
The duration the drug was taken, years	0.72	0.19	0.50–1.04	0.079
Disease course	1.57	0.23	1.01–2.45	0.046

*SARS-CoV-2* severe acute respiratory syndrome coronavirus type-2, *pwMS* patients with multiple sclerosis, *CI* confidence interval

Patients with SARS-CoV-2 infection had symptoms such as fever, sore throat, limb pain, diarrhea, cough, fatigue, and dyspnea; and most patients had a fever (89.8%). None of the above patients were admitted to the hospital, and there were no deaths.

### Variables related to SARS-CoV-2-driven self-conscious aggravation of MS post-infection

Table 3 shows the demographic and clinical data of the 117 patients with no obvious changes in their original condition after SARS-CoV-2 infection and 20 patients who felt that their original condition was worsening. 20 patients (14.6%) had aggravated symptoms post-infection, and 7 patients (5.1%) had a relapse of MS. There were no significant differences in gender, age, disease course, vaccination, immunotherapy, or immunotherapy time among the above patients.

**Table 3:** Differences between aggravated and unaggravated patients after SARS-CoV-2-infection

	Aggravated patients (*N* = 20)	Unaggravated patients (*N* = 117)	*P* Value
Gender ^a^			
Male	5 (25.0)	35 (29.9)	0.655
Female	15 (75.0)	82 (70.1)	
Age, years ^b^	38.3 ± 10.9	38.4 ± 11.4	0.922
Drug type ^a^			
Unmedicated	1 (5.0)	14 (12.0)	0.837
Glucocorticoid drugs	0	3 (2.6)	
Siponimod or Fingolimod	10 (50.0)	41 (35.0)	
Ofatumumab	1 (5.0)	4 (3.4)	
Dimethyl fumarate	0	3 (2.6)	
Teriflunomide	8 (40.0)	50 (42.7)	
Rituximab	0	2 (1.7)	
The duration the drug was taken, years ^b^	1.8 ± 1.0	2.0 ± 1.8	0.833
≤ 1 ^a^	8 (40.0)	60 (51.3)	0.469
> 1 ^a^	12 (60.0)	57 (48.7)	
Vaccination ^a^			
Unvaccinated	15 (75.0)	76 (65.0)	0.379
Vaccinated, doses	5 (25.5)	41 (35.0)	
1	0	5 (4.3)	0.888
2	3 (15.0)	17 (14.5)	
3	2 (10.0)	19 (16.2)	
Disease course, years ^a^			
≤ 5	14 (70.0)	68 (58.1)	0.751
5–10	4 (20.0)	29 (24.8)	
10–15	1 (5.0)	13 (11.1)	
> 15	1 (5.0)	7 (6.0)	

*SARS-CoV-2* severe acute respiratory syndrome coronavirus type-2

^a^ Values given as *N* (%)

^b^ Values given as mean ± standard deviation

The average age of 7 patients with relapse was 38.3 ± 9.1 years old, five (71.4%) were female, five (71.4%) were treated with teriflunomide, five (71.4%) were treated for > 1 year, and four (57.1%) were not vaccinated with COVID-19.

### Variables related to self-conscious exacerbation of MS post-SARS-CoV-2-infection

Table 4 summarises the demographic and clinical data of the 20 patients with self-conscious aggravation of their original condition after SARS-CoV-2 infection. There were significant behavioral differences among the 20 patients with aggravation of their original condition after SARS-CoV-2 infection. These patients were older (mean 44.1 ± 8.3 vs 31.2 ± 9.7, *P* = 0.008), but there was no significant difference in gender, disease course, vaccination, immunotherapy and immunotherapy time. Of the 20 patients who were aware of the aggravation of their original condition, two (10.0%) had behavioral, cognitive, and psychological aggravation, and eight (40.0%) had two or more aggravating manifestations.

**Table 4:** Differences of aggravated patients after SARS-CoV-2-positive in behavior, cognition, psychology and other

	Behavior			Cognition			Psychology			Other		
	Positive (*N* = 11)	Negative (*N* = 9)	*P* Value	Positive (*N* = 9)	Negative (*N* = 11)	*P* Value	Positive (*N* = 7)	Negative (*N* = 13)	*P* Value	Positive (*N* = 5)	Negative (*N* = 15)	*P* Value
Gender ^a^												
Male	3 (27.3)	2 (22.2)	1.000	2 (22.2)	3 (27.3)	1.000	3 (42.9)	2 (15.4)	0.290	2(40.0)	3 (20.0)	0.560
Female	8 (72.7)	7 (77.8)		7 (77.8)	8 (72.7)		4 (57.1)	11 (84.6)		3(60.0)	12(80.0)	
Age, years ^b^	44.1 ± 8.3	31.2 ± 9.7	0.008	40.3 ± 10.1	36.6 ± 11.7	0.621	33.9 ± 12.7	40.7 ± 9.5	0.153	35.0 ± 9.3	39.4 ± 11.5	0.406
Drug type ^a^												
Unmedicated	1 (9.1)	0	1.000	0	1 (9.1)	1.000	0	1 (7.7)	1.000	0	1 (6.7)	1.000
DMT	10 (90.9)	9 (100.0)		9 (100.0)	10 (90.9)		7 (100.0)	12 (92.3)		5 (100.0)	14 (93.3)	
The duration the drug was taken, years ^b^	1.7 ± 1.0	1.9 ± 1.4	0.877	2.0 ± 0.9	1.0 ± 1.1	0.297	1.5 ± 1.2	2.0 ± 1.0	0.334	1.7 ± 1.0	1.8 ± 1.1	0.929
≤ 1 ^a^	5 (45.5)	3 (33.3)	0.670	3 (33.3)	5 (45.5)	0.670	4 (57.1)	4 (30.8)	0.356	2 (40.0)	6 (40.0)	1.000
> 1 ^a^	6 (54.5)	6 (66.7)		6 (66.7)	6 (54.5)		3 (42.9)	9 (69.2)		3 (60.0)	9 (60.0)	
Vaccination ^a^												
Vaccinated	3 (27.3)	2 (22.2)	1.000	2 (20.0)	3 (30.0)	1.000	2 (28.6)	3 (23.1)	1.000	3 (60.0)	2 (13.3)	0.073
Unvaccinated	8 (72.7)	7 (77.8)		8(80.0)	7(70.0)		5 (71.4)	10 (76.9)		2 (40.0)	13 (86.7)	
Disease course, years ^a^												
≤ 5	6 (54.5)	8 (88.9)	0.157	6 (66.7)	8 (72.7)	1.000	5 (71.4)	9 (69.2)	1.000	3 (60.0)	11 (73.3)	0.613
> 5	5 (45.5)	1 (11.1)		3 (33.3)	3 (27.3)		2 (28.6)	4 (30.8)		2 (40.0)	4 (26.7)	

SARS-CoV-2, severe acute respiratory syndrome coronavirus type-2; DMT, disease-modifying therapy

^a^ Values given as *N* (%)

^b^ Values given as mean ± standard deviation

## Discussion

This epidemiological investigation of MS and COVID-19 in northern China provides several noteworthy observations: (1) A shorter course of pwMS was correlated with SARS-CoV-2 infection risk and (2) The pwMS with aggravated behavior post-SARS-CoV-2-infection were older. (3) The clinical outcomes of pwMS diagnosed with SARS-CoV-2 infection were favorable with no hospitalization and mortality. (4) The vaccination rate among pwMS was significantly lower than that of the general population. (5) Vaccination and immunotherapy had no effect on SARS-CoV-2 infection and the self-conscious aggravation of the original condition and manifestation.

The incidence of SARS-CoV-2 infection in pwMS remains unclear compared to the general population. In our cohort, there were differences in the disease course between SARS-CoV-2 infected patients and non-SARS-CoV-2 infected patients. A shorter disease course correlated with a heightened infection risk in pwMS. Generally, as the disease progresses, the degree of disability in pwMS increases. A UK study suggested that patients with older age, higher disability, and highly effective DMT were more inclined to adopt protecting behavior against SARS-CoV-2 infection [13]. This may explain the higher SARS-CoV-2 infection rate observed in patients with a shorter disease course in our study. Nevertheless, we cannot completely discount the impact of other factors on SARS-CoV-2 infection incidence, including comorbid diseases, MS type and other modifiable risk factors [14]. A retrospective study from Italy [15] evaluating modifiable risk factors associated with COVID-19 in pwMS suggests that higher Vitamin D levels and teleworking may prevent the risk of unwanted infections in pwMS. This also provides a more practical strategy for the management of pwMS during pandemic.

Our data showed that patients with aggravated behavioral manifestations after SARS-CoV-2 infection were older than those without such manifestations. Previous studies [16–18] have indicated a correlation between age in MS cohorts and the prognosis of COVID-19. However, few studies have reported whether age is related to behavior, cognition, psychology, or other specific aspects of post-SARS-CoV-2 infection. We speculate that, in general, older patients often experience a longer disease course and higher physical disability degree, and are more likely to have aggravated behavioral manifestations post-infection. A large case–control study from Italy [19] found that COVID-19 had no significant effect on disease activity, disease duration, and cognitive function in pwMS 18 to 24 months after infection, which differs from our findings. Differences in the populations studied, sample sizes, differences in viral strains, epidemic prevention policies adopted by regions, and differences in protective behaviors adopted by individuals during the pandemic may explain the differences in conclusions. Firstly, the populations of the two studies are inconsistent. There may be differences in the performance of different populations in COVID-19 pandemic. Secondly, the sample size may be one of the reasons for the inconsistency of the research results. Furthermore, SARS-CoV-2, as a RNA virus, is easy to mutate. At present, there are numerous kinds of variants worldwide, and the prevalent virus variants vary among different countries and regions. According to the data of China Centers for Disease Control and Prevention [11], the predominant strain of SARS-CoV-2 in the three northern provinces of China during the survey period was OmicronBA.5.2, while Delta variant (B.1.167.2 lineage) was one of the predominant strains in Italy during the above-mentioned research period [20]. Lastly, due to the differences in the development process of COVID-19 in different parts of the world, the differences in epidemic prevention policies and individual protection behaviors adopted by different regions may also lead to differences in the survey results of different populations. Further investigation is required to ascertain the specific reasons.

While infection may lead to relapse in pwMS, this information has rarely been reported in other studies, leaving the exact impact of the pandemic on clinical disease activity unclear [21]. Our data showed no statistically significant differences between patients with aggravated post-SARS-CoV-2 infection symptoms and those without aggravation. The patients infected by SARS-CoV-2 were not hospitalized, received satisfactory clinical management and with no deaths, and both DMT-accepting and non-DMT-accepting patients had good conversion rules, which is not obviously different from the general population. Studies [22] have shown that MS disease activity did not increase during the pandemic. However, its significant decrease may be attributed to underreporting rather than a real decrease in disease activity during the pandemic.

As of 31 March 2023, various vaccines against SARS-CoV-2 have been developed worldwide. Vaccination types for the Chinese population mainly included inactivated, adenovirus vector, and recombinant protein vaccines. Data from the Chinese Center for Disease Control and Prevention [11] showed that, as of 31 March 2023, a total of 1.310459 billion individuals in China had been vaccinated, with 1.277044 billion people having been vaccinated throughout the course, and 827.658 million people completing the first dose of enhanced vaccination. The coverage rates of the first dose and the entire course of the vaccination in the whole population were 93.0% and 90.6%, respectively. In the cohort of pwMS, 55 (33.5%) were vaccinated with the COVID-19 vaccine, showing a significantly lower vaccination rate than that in the general Chinese population. A UK study [23] showed that most pwMS were willing to be vaccinated after receiving the COVID-19 vaccine, and the main reasons for not receiving the vaccine include safety considerations and lack of information. A study on the COVID-19 vaccine in North America [24] supported this conclusion. In our cohort, vaccination had no statistical effect on SARS-CoV-2-infection risk and prognosis. Studies [25, 26] have shown that hospitalization, severe illness, and death decreased significantly after receiving the COVID-19 vaccination in the general population. Similarly, there is a study [27] to demonstrate that the COVID-19 vaccine proves safe for pwMS and is not associated with an increased risk of relapse activity.

DMT may also be associated with SARS-CoV-2 infections. Most treatments for MS are based on the principle of immune system suppression, which may make these patients more vulnerable to SARS-CoV-2 infection. Some reports [28–30] have suggested that anti-CD-20 B-cell depletion agents or sphingosine-1-phosphate receptor modulators can reduce the humoral immune response to SARS-CoV-2, while the cellular immune response may be retained or even partially enhanced. Consequently, DMT may affect the response of pwMS to SARS-CoV-2 infections. A systematic review of 87 studies [31] indicated that the use of DMT appeared to be generally safe, with no significant increase in the risk of a poor prognosis for COVID-19. However, there may be signals suggesting that B-cell depletion therapy slightly exacerbates COVID-19 infection. A comprehensive analysis of Italian and French cohorts [32] suggested that a significant association between anti-CD20 drugs and COVID-19 severity with the risk increasing with treatment duration. A meta-analysis [8] showed that patients treated with rituximab had a higher severe COVID-19 risk than those who received other treatments. However, in our cohort, there were no significant differences in the type and time of receiving immunotherapy for SARS-CoV-2 infection or MS prognosis. Although COVID-19 patients received a longer period of immunotherapy than non-infected patients, the difference was not statistically significant (*p* = 0.056). This result is consistent with that of a study from Chile [17] that reported the possible impact of complication status, age, male gender, and higher Expanded Disability Status Scale on COVID-19 prognosis but was not significantly related to any particular DMT. A national study from China [33], including 361 pwMS who were from 22 of 23 provinces, 4 of 5 autonomous regions, and 3 of 4 municipalities across China, showed that none of the pwMS infected with COVID-19 during the pandemic under precautions, whereas patients who interrupted treatment experienced a significantly higher annualized relapse rate during the pandemic compared to pre-pandemic levels. In summary, the risk of MS exacerbations due to treatment interruption may outweigh the risk of COVID-19 infection, emphasizing the importance of maintaining treatment of MS. However, caution is when using advised B-cell-depleting drugs [34].

There are limitations in our study. Firstly, the sample size in this study was relatively small, which may limit the generalizability of our findings. Secondly, we employed an online self-administered questionnaire, potential selection bias caused by self-reported data from participants cannot be ruled out. In the cohort of this study, 94 (57.3%) people had a disease duration of ≤ 5 years, and 83.5% of patients were infected with SARS-CoV-2, which may be related to selection bias. And the relatively low response rate may also result in selection bias. Thirdly, due to the limited number of patients with aggravation or relapse post-SARS-CoV-2 infection, we could only build our experience based on a smaller group of patients about this part of the data (Table 4). Therefore, this portion of data should be treated with caution. Lastly, concerning DMT, having only been on the market in China for one year as of the time of the questionnaire, Ofatumumab was the first and sole Chinese National Medical Products Administration-approved B-cell depleting agent indicated for MS therapy. This may have some implications for the data analysis related to immunotherapy.

## Conclusion

In this cross-sectional study, a shorter course of diseases is independently associated with an increased risk of SARS-CoV-2 infection, and the risk factor for worsening disability is age. It seems to be safe and necessary to use DMT during the pandemic, however, the use of B cell-depletion agents should be approached with caution. Therefore, pwMS should be recommended to take personal protective measures to reduce the risk of exposure and receive standardized treatment. The results of this study could help inform the development of management strategies for current COVID-19 and long-term or future pandemics.

## Data Availability

The data that support the findings of this study are available on request from the corresponding author. The data are not publicly available due to privacy or ethical restrictions.
